# Molecular Insight into the Acryloyl-CoA Hydration by AcuH for Acrylate Detoxification in Dimethylsulfoniopropionate-Catabolizing Bacteria

**DOI:** 10.3389/fmicb.2017.02034

**Published:** 2017-10-17

**Authors:** Hai-Yan Cao, Peng Wang, Fei Xu, Ping-Yi Li, Bin-Bin Xie, Qi-Long Qin, Yu-Zhong Zhang, Chun-Yang Li, Xiu-Lan Chen

**Affiliations:** ^1^State Key Laboratory of Microbial Technology, Marine Biotechnology Research Center, Institute of Marine Science and Technology, Shandong University, Jinan, China; ^2^Laboratory for Marine Biology and Biotechnology, Qingdao National Laboratory for Marine Science and Technology, Qingdao, China

**Keywords:** DMSP, acrylate, detoxification, acryloyl-CoA hydratase, catalytic mechanism

## Abstract

Microbial cleavage of dimethylsulfoniopropionate (DMSP) producing dimethyl sulfide (DMS) and acrylate is an important step in global sulfur cycling. Acrylate is toxic for cells, and thus should be metabolized effectively for detoxification. There are two proposed pathways for acrylate metabolism in DMSP-catabolizing bacteria, the AcuN-AcuK pathway and the PrpE-AcuI pathway. AcuH is an acryloyl-CoA hydratase in DMSP-catabolizing bacteria and can catalyze the hydration of toxic acryloyl-CoA to produce 3-hydroxypropionyl-CoA (3-HP-CoA) in both the AcuN-AcuK pathway and the side path of the PrpE-AcuI pathway. However, the structure and catalytic mechanism of AcuH remain unknown. Here, we cloned a putative *acuH* gene from *Roseovarius nubinhibens* ISM, a typical DMSP-catabolizing bacterium, and expressed it (*Rd*AcuH) in *Escherichia coli*. The activity of *Rd*AcuH toward acryloyl-CoA was detected by liquid chromatography-mass spectrometry (LC-MS), which suggests that *Rd*AcuH is a functional acryloyl-CoA hydratase. Then we solved the crystal structure of *Rd*AcuH. Each asymmetric unit in the crystal of *Rd*AcuH contains a dimer of trimers and each *Rd*AcuH monomer contains an N-terminal domain (NTD) and a C-terminal domain (CTD). There are three active centers in each trimer and each active center is located between the NTD of a subunit and the CTD of the neighboring subunit. Site-directed mutagenesis analysis indicates that two highly conserved glutamates, Glu112 and Glu132, in the active center are essential for catalysis. Based on our results and previous research, we analyzed the catalytic mechanism of AcuH to hydrate acryloyl-CoA, in which Glu132 acts as the catalytic base. This study sheds light on the mechanism of acrylate detoxification in DMSP-catabolizing bacteria.

## Introduction

Dimethylsulfoniopropionate (DMSP), a secondary metabolite of marine phytoplankton, macroalgae and some angiosperms, is a major participant in the global sulfur and carbon cycles (Kiene et al., 2000; Stefels et al., 2007; Curson et al., 2011; Li et al., 2014; Wang et al., 2015). DMSP can be produced on a scale of 10^3^ Tg each year and accounts for approximately 10% of the total organic carbon in some places of the seawater (Archer et al., 2001; Simo et al., 2002). The marine Roseobacter clade and the SAR11 clade are the principal groups of bacteria involved in DMSP degradation (Curson et al., 2011). They metabolize DMSP via two general pathways, the demethylation pathway and the cleavage pathway (Curson et al., 2011; Johnston et al., 2016). Every year, approximately 300 Tg of dimethyl sulfide (DMS) and equal mole of acrylate are produced by microbial cleavage (Lovelock et al., 1972; Curson et al., 2011).

Acrylate is an important carbon source in the ocean and can be used as a sole carbon source for some bacteria (Yoch, 2002; Todd et al., 2010; Curson et al., 2014). However, acrylate and its metabolite acryloyl-CoA are toxic when they are accumulated in cells (Todd et al., 2010, 2012; Reisch et al., 2013). Thus, rapid metabolism of acrylate and acryloyl-CoA is essential for the survival of the DMSP-catabolizing bacteria.

**FIGURE 1:**
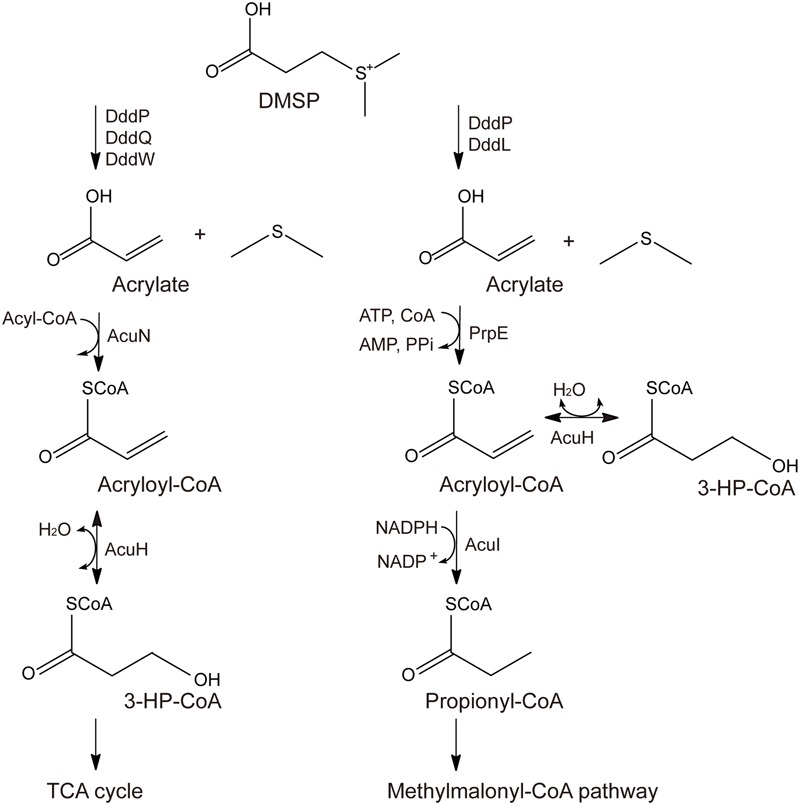
Proposed metabolic pathways for acrylate detoxification. The left is the AcuN-AcuK pathway in *Halomonas* sp. HTNK1 (Curson et al., 2011; Reisch et al., 2013), and the right is the PrpE-AcuI pathway in *Ruegeria pomeroyi* DSS-3 (Curson et al., 2011; Reisch et al., 2013).

Two different pathways for acrylate metabolism and detoxification in DMSP-catabolizing bacteria have been proposed, the AcuN-AcuK pathway and the PrpE-AcuI pathway (Curson et al., 2011; Reisch et al., 2013; Wang et al., 2017). In the AcuN-AcuK pathway, acrylate is metabolized to 3-hydroxypropionyl-CoA (3-HP-CoA) by AcuN and AcuH, which is also named AcuK (Curson et al., 2011), and finally metabolized through the tricarboxylic acid (TCA) cycle (**Figure 1**). In the PrpE-AcuI pathway, acrylate is metabolized to propionyl-CoA by PrpE and AcuI and finally metabolized through the methymalonyl-CoA pathway (**Figure 1**) (Curson et al., 2011; Reisch et al., 2013; Bullock et al., 2017).

In acrylate metabolism and detoxification, AcuH is functional in both the AcuN-AcuK pathway and the PrpE-AcuI pathway (Curson et al., 2011; Reisch et al., 2013; Bullock et al., 2017) (**Figure 1**). It can catalyze acryloyl-CoA hydration in the AcuN-AcuK pathway and the branching of the PrpE-AcuI pathway (Curson et al., 2011; Reisch et al., 2013; Bullock et al., 2017) (**Figure 1**). AcuH belongs to the enoyl-CoA hydratase family and is first identified from *Halomonas* sp. HTNK1, a γ-proteobacterium that can grow on acrylate as sole carbon source (Todd et al., 2010). Enoyl-CoA hydratase usually catalyzes the second step of the β-oxidation of fatty acid degradation (Bahnson et al., 2002; Nelson and Cox, 2005). Enoyl-CoA hydratases can use various enoyl-CoA with different lengths as substrates, such as crotonyl-CoA, methacrylyl-CoA and tiglyl-CoA. Enoyl-CoA hydratases from different sources, such as bovine liver and rat liver, have been characterized (Steinman and Hill, 1975; Furuta et al., 1980). The mechanisms of some enoyl-CoA hydratases, such as an enoyl-CoA hydratase from the liver of *Rattus norvegicus* and EchA6 from *Mycobacterium tuberculosis* have been further studied (Bahnson et al., 2002; Cox et al., 2016). However, the structure of AcuH and its catalytic mechanism to catalyze acryloyl-CoA hydration in DMSP-catabolizing bacteria remains unknown.

*Roseovarius nubinhibens* ISM, a member of the marine Roseobacter clade, is an aerobic bacterium that is capable of degrading DMSP. It was isolated from the caribbean sea seawater (Fuhrman et al., 1994) and has been whole-genome sequenced. Its genome sequence has been deposited in the NCBI database (GCA_000152625.1), which contains a putative AcuH (WP_009813188.1). In order to insight the structure and catalytic mechanism of AcuH in acrylate mechanism pathway in DMSP-catabolizing bacteria, in this study, we cloned the putative *acuH* gene from *Roseovarius nubinhibens* ISM and heterologously expressed it in *Escherichia coli* BL21 (DE3). The recombinant AcuH was named *Rd*AcuH. We detected the acryloyl-CoA hydration activity of *Rd*AcuH and solved its three-dimensional structure. Then, by structural and site-directed mutagenesis analyses, we found two conserved glutamates, Glu112 and Glu132, are key residues involved in catalysis. Finally, based on our structural and mutagenesis analyses, we proposed a catalytic mechanism for *Rd*AcuH hydrating acryloyl-CoA to produce 3-HP-CoA. The results offer a better understanding for the process and molecular mechanism of acrylate detoxification in DMSP-catabolizing bacteria.

## Materials and Methods

### Bacterial Strains and Growth Conditions

*Roseovarius nubinhibens* ISM was purchased from the Leibniz Institute DSMZ-German Collection of Microorganisms and Cell Cultures, and was cultured in 974 medium at 30°C for 2 days according to the provided protocol^1^. *E. coli* DH5α and *E. coli* BL21 (DE3) were all purchased from TransGen Biotech company (China) and were grown in Luria-Bertani (LB) medium at 37°C. *E. coli* DH5α and *E. coli* BL21 (DE3) were used for gene cloning and gene expression, respectively.

### Gene Cloning, Site-Directed Mutation, Protein Expression and Purification

The Rd*acuH* gene was amplified from the genome of *Roseovarius nubinhibens* ISM by PCR using *FastPfu* DNA polymerase. PCR primers were designed with the *Nde*I and *Xho*I restriction sites. The amplified Rd*acuH* is recombined to the vector pET-22b. *FastPfu* DNA polymerase and the vector pET-22b were purchased from TransGen Biotech (China) and Novagen company (Germany), respectively.

All site-directed mutations were introduced using overlap PCR and verified by sequencing. The constructed recombinant plasmids, including the wild type Rd*acuH* and its mutants were transferred into *E. coli* BL21 (DE3) for expression. The cells were cultured in LB medium with 0.1 mg/ml ampicillin at 37°C to an OD_600_ of 1.0-1.2. Then the culture was induced at 20°C overnight with 0.2 mM isopropyl-β-D-thiogalactopyranoside (IPTG). Wild type *Rd*AcuH and its mutants were purified by affinity chromatography on a Ni^2+^-NTA column (GE healthcare, United States), and then fractionated by anion exchange chromatography on a Source 15Q column (GE healthcare, United States) and gel filtration on a Superdex G200 column (GE healthcare, United States).

The *prpE* gene was amplified from the genomic DNA of *Dinoroseobacter shibae* DFL 12. Expression and purification of PrpE were conducted with the same methods as *Rd*AcuH described above.

### Enzyme Assays

PrpE, a propionate-CoA ligase from *D. shibae* DFL 12, was used to produce acryloyl-CoA. Reaction system of PrpE contained 0.5 mM acrylate, 0.5 mM coenzyme A (CoA), 0.5 mM adenosine triphosphate (ATP), 5 mM MgCl_2_, 100 mM Tris-HCl (pH 8.0) and 10 μM recombinant PrpE. After a 2-h reaction at 37°C, *Rd*AcuH was added to the mixture at a final concentration of 1 μM. Hydrochloric acid was added to a final concentration of 1 mM after 5 min to terminate the reaction. The enzyme activity of *Rd*AcuH was measured by determining the production of 3-HP-CoA using liquid chromatography-mass spectrometry (LC-MS). Components of the reaction system were separated on a reversed-phase C_18_ column (4.6 × 250 mm, 5 μm particle size; Waters, United States) connected to a high performance liquid chromatography (HPLC) system (Dionex, United States). The detection wavelength was 260 nm. The samples were eluted with a linear gradient of 1-20% (v/v) acetonitrile in 20 mM ammonium acetate (pH 5.5) over 15 min at a flow rate of 1 ml/min. The injection volume was 5 μl and the column temperature was 25°C. The HPLC system was coupled to an impact HD mass spectrometer (Bruker, Germany) for molecular weight (MW) determination.

### Crystal Screening and Data Collection

Crystal Screen Reagent Kits (Hampton, VA, United States; Qiagen, Germany) were used for crystal screening. The concentration of the purified protein was approximately 10 mg/ml. The initial buffer for crystallization contained 0.1 M sodium acetate (pH 5.0), 15% (v/v) 2-methyl-1,3-propanediol and 2% (w/v) PEG 4000. Then the crystals were optimized using the hanging drop vapor diffusion method at 20°C (the ratio of protein to precipitant was 1:1). The optimal buffer for crystallization contained 0.1 M sodium acetate (pH 5.2), 15% (v/v) 2-methyl-1,3-propanediol and 2.2% (w/v) PEG 4000. Precipitant with 15% glycerol was used as cryoprotection buffer and the crystals were frozen in liquid nitrogen. X-ray diffraction data were collected at the Shanghai Synchrotron Radiation Facility beamline BL17U1 (Wang et al., 2016). The wavelength was 0.9791 Å. Diffraction data were collected using an ADSC Q315r CCD detector (Poway, CA, United States) at 100 K and processed with the HKL2000 package (Ducruix and Giegé, 1999).

### Structure Determination and Refinement

The structure of *Rd*AcuH was solved by molecular replacement using Phaser (Potterton et al., 2003). An enoyl-CoA hydratase (PDB ID code: 3H81) was used as the searching model. The automated model building was performed with *ARP*/*wARP* (Langer et al., 2008). Manual model refinement was performed with COOT (Emsley et al., 2010) and PHENIX (Emsley et al., 2010). All the structural figures were generated using the PyMOL program^2^.

### Circular Dichroism (CD) Spectroscopy

Circular dichroism (CD) spectra were collected from 250 to 200 nm on a J-810 spectropolarimeter (Jasco, Japan) at 25°C. A quartz cuvette with a path length of 0.1 cm was used. All proteins were adjusted to a final concentration of 0.2 mg/ml in 10 mM Tris-HCl (pH 8.0).

### Dynamic Light Scattering (DLS)

Dynamic light scattering experiment was performed on a Dynapro Titan TC (Wyatt Technology, United States) at 4°C using 20 μl (2.5 mg/ml) *Rd*AcuH in a buffer containing 10 mM Tris-HCl (pH 8.0) and 100 mM NaCl. Data were analyzed with dynamics 7 1.0 software.

### Accession Numbers

The structures of *Rd*AcuH was deposited in the Protein Data Bank under the accession codes 5XZD.

## Results

### Gene Cloning and Sequence Analysis of *Rd*AcuH

The putative *acuH* gene, Rd*acuH* (GI:497498990), was identified from the genome of *Roseovarius nubinhibens* ISM. The ORF of Rd*acuH* contains 777 bp, encoding a protein (*Rd*AcuH) of 258 amino acid residues. The MW and isoelectric point (pI) of *Rd*AcuH monomer is predicted to be 28.3 kDa and 5.07, respectively, using the compute pI/Mw tool^3^ (Gasteiger et al., 2005). In addition, *Rd*AcuH contains no signal peptide based on the prediction by SignalP 4.0 Server^4^ (Petersen et al., 2011), suggesting that *Rd*AcuH is an intracellular protein.

**FIGURE 2:**
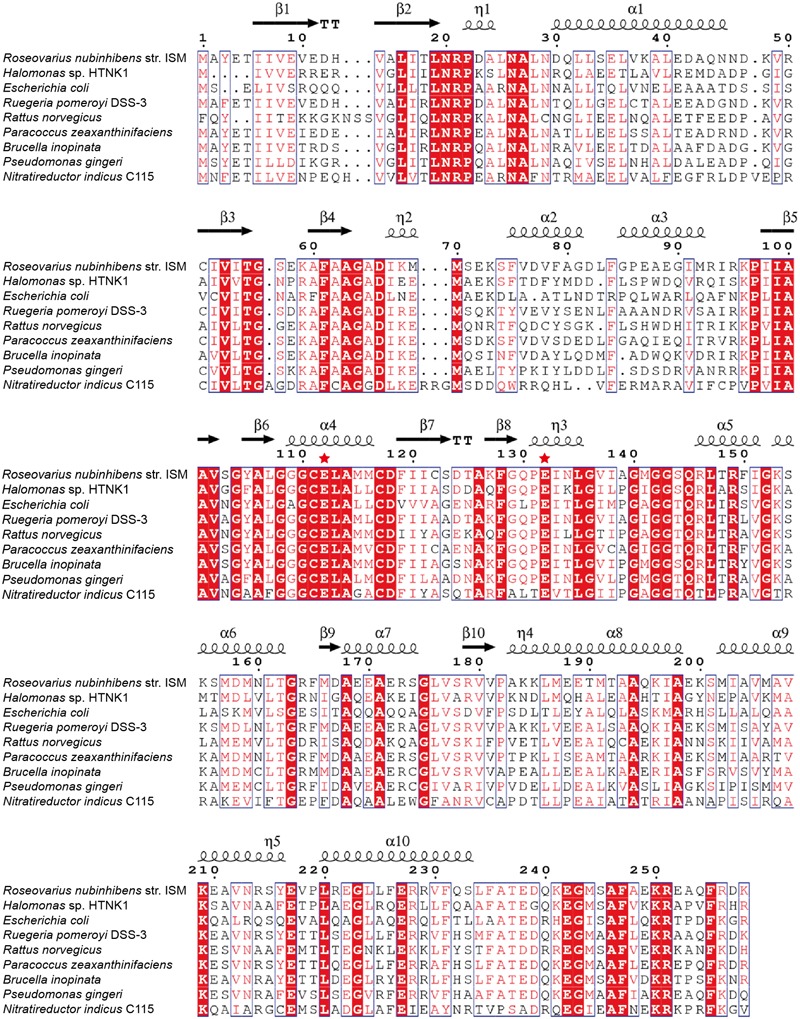
Sequence alignment of *Rd*AcuH with other enoyl-CoA hydratases. Red filled boxes represent identical residues. The putative catalytic residues are marked by red stars. Picture was generated using ESPript 3.0 (Robert and Gouet, 2014).

Conserved domain analysis (Marchlerbauer et al., 2015) suggests that *Rd*AcuH belongs to the crotonase superfamily (1.07e^-74^) and is predicted as an enoyl-CoA hydratase (6.64e^-150^). *Rd*AcuH presents 56 and 79% identities to the known AcuHs from *H.* sp. HTNK1 and *Ruegeria pomeroyi* DSS-3, respectively (Todd et al., 2010). In addition, *E. coli*, a model prokaryote, and *R. norvegicus*, a model vertebrate, also have several *Rd*AcuH homologues. The most similar sequences from *E. coli* and *R. norvegicus* show 45% and 53% identities to *Rd*AcuH, respectively (**Figure 2**).

### Expression and Purification of *Rd*AcuH

**FIGURE 3:**
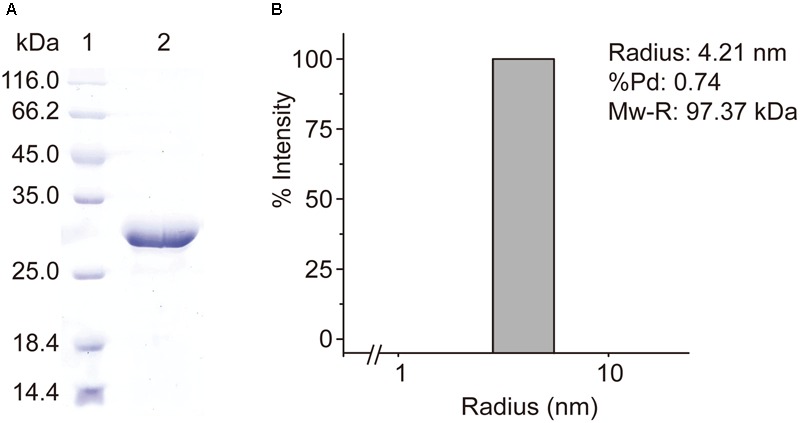
Purification and characterization of *Rd*AcuH. **(A)** SDS-PAGE analysis of the purified *Rd*AcuH protein, showing an apparent MW of about 28 kDa. **(B)** DLS analysis of the molecular weight of *Rd*AcuH in solution. The %Pd is 0.74, indicating that it has high uniformity. The estimated molar mass is 97.37 kDa, indicating that it is a trimer.

Rd*acuH* was recombined to vector pET-22b and the recombinant *Rd*AcuH was expressed in *E. coli* BL21 (DE3). *Rd*AcuH was purified to electrophoretic homogeneity by a three-step purification. SDS-PAGE analysis of the purified enzyme showed that the apparent MW of *Rd*AcuH is about 28 kDa (**Figure 3A**), which is consistent with the predicted MW of 28.3 kDa. DLS analysis revealed that the estimated MW of *Rd*AcuH in solution is 97.37 kDa (**Figure 3B**), which is approximately three times of the apparent MW of *Rd*AcuH, suggesting that *Rd*AcuH is a trimer in solution. The %Pd is 0.74, indicating that the sample has high uniformity and the result is reliable.

### Activity Assay of *Rd*AcuH

It has been reported that acryloyl-CoA, the substrate of *Rd*AcuH, was synthesized by chemical methods (Stadtman, 1957). However, during the synthesis process, we found that the production was very low and that acryloyl-CoA was very unstable. So we used PrpE (**Figure 1**), an acrylate-CoA ligase, to biologically produce acryloyl-CoA. The *prpE* gene was cloned from *D. shibae* DFL 12, a *Roseobacter* that can also metabolize DMSP (Dickschat et al., 2010). After a 2-h reaction of PrpE, *Rd*AcuH was added to the reaction system of PrpE for the enzyme activity detection of *Rd*AcuH (see “Materials and Methods” for details).

**FIGURE 4:**
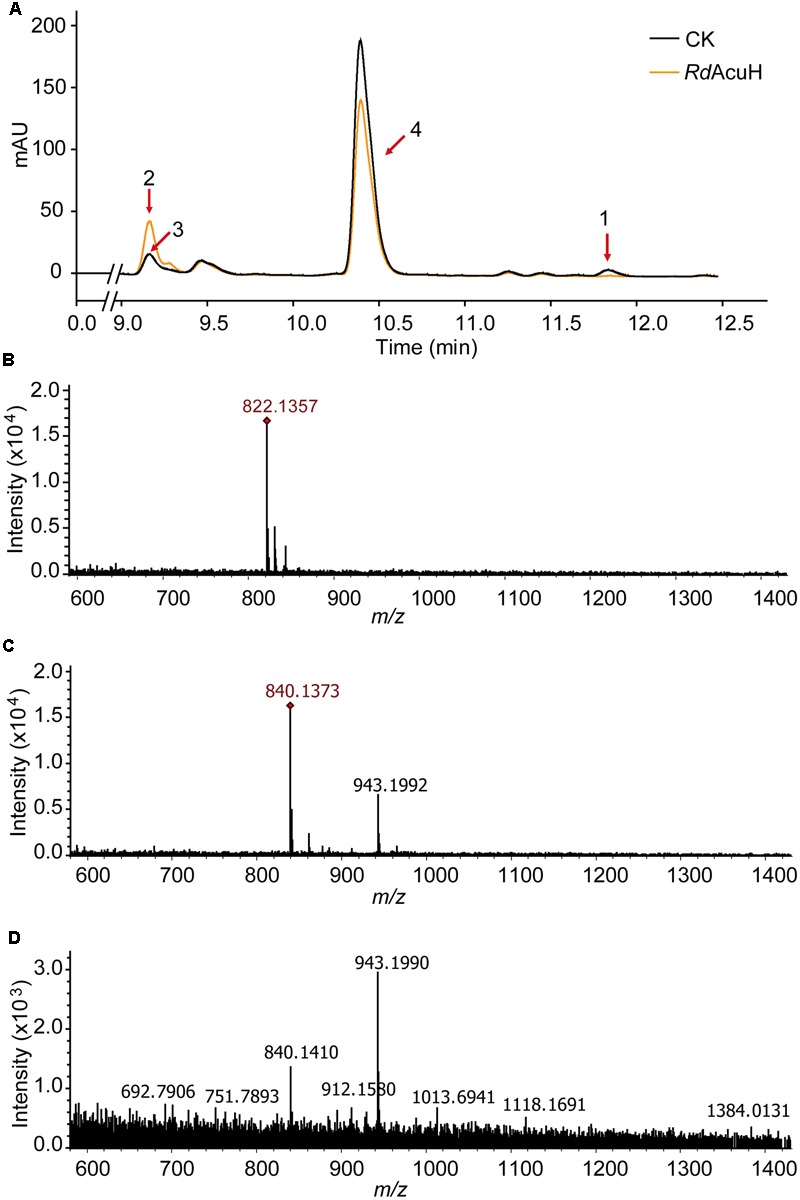
Enzymatic activity detection of *Rd*AcuH. **(A)** Enzyme assay of *Rd*AcuH by HPLC. **(B)** LC-MS detection of the components of peak 1. The MW of the main compound is 822.1357, which is equal to the MW of acryloyl-CoA, indicating that it is acryloyl-CoA. **(C)** LC-MS detection of the components of peak 2. The MW of the main compound is 840.1351, which is equal to the MW of 3-HP-CoA, indicating that it is 3-HP-CoA. **(D)** LC-MS detection of peak 3. Complicate components including a little 3-HP-CoA were detected.

LC-MS were used to detect the activity of *Rd*AcuH. The results of liquid chromatography showed that a peak vanished (peak1) and another peak (peak2) increased after a 5-min reaction of *Rd*AcuH (**Figure 4A**). The MW of the component of the vanished peak is 822.1357, which is equal to the MW of acryloyl-CoA (**Figures 4A,B**) and the MW of the component of the increased peak is 840.1351, which is equal to the MW of 3-HP-CoA (**Figures 4A,C**). This indicated that, during the reaction, acryloyl-CoA was hydrated by *Rd*AcuH and 3-HP-CoA was produced. There was also a small peak (peak 3) in the same position of the increased peak in the control group, and a little 3-HP-CoA was detected from this peak (**Figures 4A,D**), probably because acryloyl-CoA is unstable and trace of acryloyl-CoA could be hydrated to 3-HP-CoA spontaneously. In addition, peak 4 is an impurity peak with complex composition and no acryloyl-CoA or 3-HP-CoA was detected. The activity assay results showed that *Rd*AcuH has acryloyl-CoA hydratase activity and can catalyze acryloyl-CoA to 3-HP-CoA.

### Crystallization and Structure Determination of *Rd*AcuH

**FIGURE 5:**
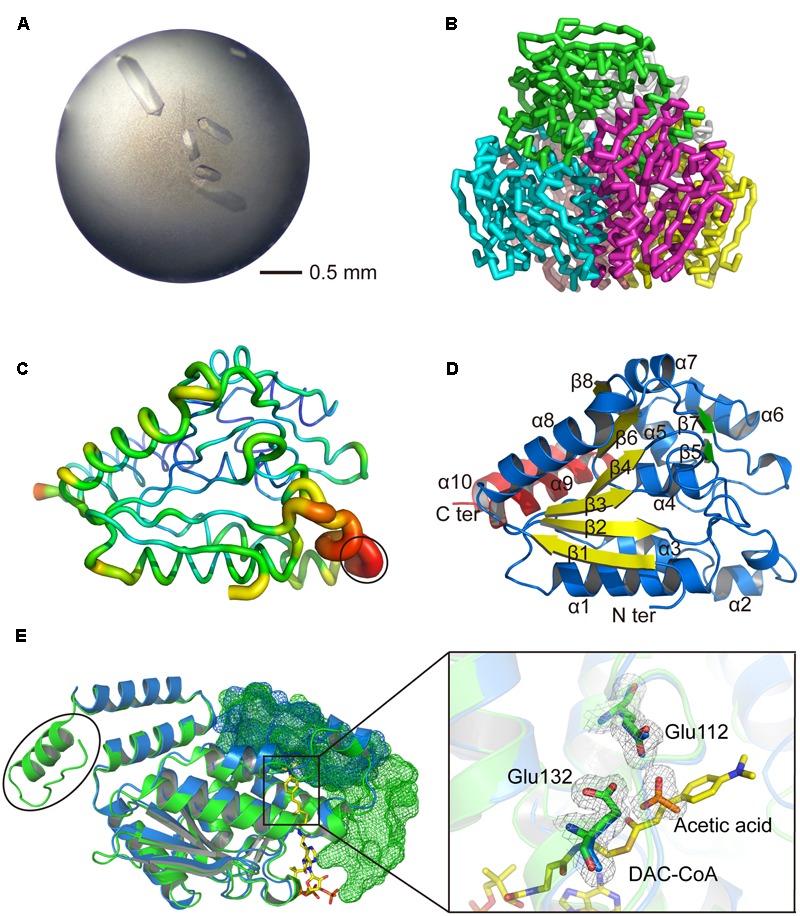
Structural analysis of *Rd*AcuH. **(A)** Optimized crystal of *Rd*AcuH. **(B)** Overall structure of *Rd*AcuH. The structure is made up of two stacked trimers. The three subunits in the up trimer are colored in blue, green and magentas, respectively. The other three subunits in the down trimer are colored in pink, gray and yellow, respectively. **(C)** B-factor view of *Rd*AcuH monomer (chain A). The B-factor increases from the cool colors to the warm colors and the flexible loop of *Rd*AcuH is circled out. **(D)** Cartoon view of *Rd*AcuH monomer. The two perpendicular β-sheets are colored in yellow and green, respectively, and the CTD is colored in red. **(E)** Structural alignment of *Rd*AcuH monomer and the monomer of the enoyl-CoA hydratase from *R. norvegicus* (PDB code: 1EY3). *Rd*AcuH is colored in blue and the enoyl-CoA hydratase from *R. norvegicus* is colored in green. The CTD of the neighboring subunit of the *Rd*AcuH monomer and the CTD of the neighboring subunit of the monomer of enoyl-CoA hydratase from *R. norvegicus* are all shown in mesh views. The last 23 residues of the enoyl-CoA hydratase from *R. norvegicus* are circled out. Glu144 and Glu164 of *Rd*AcuH are colored in blue and the acetic acid in *Rd*AcuH is colored in orange. Their 2*Fo*-*Fc* electron density maps are shown at 1.0 σ. In the structure of the enoyl-CoA hydratase from *R. norvegicus*, Glu144 and Glu164 are colored in green and DAC-CoA is colored in yellow. DAC-CoA represent the possible position of acryloyl-CoA of *Rd*AcuH.

To investigate the structure and catalytic mechanism, *Rd*AcuH was crystallized. Shuttle-shaped crystals of *Rd*AcuH grew to a size of 0.8 × 0.25 × 0.25 mm in 2 days (**Figure 5A**). The X-ray diffraction data of the crystals were collected with the wavelength of 0.9791 Å. After data integration, the crystal was identified in the space group of *C*121. The unit cell of the crystal is *a* = 127.81 Å, *b* = 119.453 Å, *c* = 120.276 Å, and α = 90°, β = 102.769°, γ = 90°, and the completeness of the data is 99.34%. Data redundancy is high enough for structure determination.

A similarity search in Protein Data Bank (PDB)^5^ (Berman et al., 2000) revealed that *Rd*AcuH shares 53% sequence identity with a typical enoyl-CoA hydratase from *R. norvegicus* (PDB code: 1EY3) (covering 100% of the sequence). With the enoyl-CoA hydratase from *R. norvegicus* as a model, the structure of *Rd*AcuH was solved at the resolution of 1.9 Å. After refinement, the structure of *Rd*AcuH was refined to an *R*_work_ of 17.3%, an *R*_free_ of 19.9% and the average B-factors of 31.6 Å^2^. Ramachandran plot shows that 97.4% of the residues are in the favored regions and 2.6% of the residues are in the allowed regions. Details of data collection parameters, structure determination and refinement are summarized in **Table 1**.

**Table 1:** Data collection and refinement statistics.

Parameters	*Rd*AcuH
**Data collection statistics**	
Space group	*C* 1 2 1
Unit cell parameters	*a* = 127.81 Å *b* = 119.453 Å *c* = 120.276 Å α = 90° β = 102.769° γ = 90°
Resolution range (Å)^a^	48.38-1.9 (1.968-1.9)
Redundancy	3.6
Completeness (%)	99.34 (97.97)
*R*_merge_^b^	0.078
**Refinement statistics**	
*R*_work_^c^	0.173
*R*_free_^c^	0.199
RMSD from ideal geometry	
Bond lengths (Å)	0.008
Bond angles (°)	1.18
Ramachandran plot	
Favored (%)	97.4
Allowed (%)	2.6
Average B factors (Å^2^)	31.6

*^a^Numbers in parentheses refer to data in the highest-resolution shell. ^b^*R*_*merge*_ = Σ| Ii – Ia| /Σ(Ii), where Ii is the intensity of the measured reflection and Ia is the average intensity of all symmetry-related reflections. ^c^*R*_*work*_ = Σ|Fo – Fc|/Σ(Fo), where Fo is the observed structure factor and Fc is the calculated structure factor. *R*_*free*_ is the equivalent of *R*_*work*_, except it is calculated for a randomly chosen set of reflections (5%) from the refinement process.*

### Overall Structure of *Rd*AcuH

The overall structure of *Rd*AcuH is a dimer of trimers in an asymmetric unit of the crystal, in which three monomers associate end to end to form a tight trimer, and two trimers associate back to back to form a dimmer (**Figure 5B**). There are few strong interactions between these two trimers. Thus, the dimer should result from crystal packing.

The first 2 and the last 23 residues of each *Rd*AcuH subunit are missing in our structure. The electron densities of residues 66–76 in all chains are ambiguous except chain A in which these residues still have very high B-factors, indicating that these residues form a flexible loop (**Figure 5C**).

The overall structure of *Rd*AcuH is very similar to the structure of the enoyl-CoA hydratase from *R. norvegicus*. The RMSD between the two structures after superposition is 0.857 Å for 1239 common C_α_-atoms and 0.938 Å for all 7948 common atoms. Each *Rd*AcuH subunit contains two domains, an N-terminal domain (NTD) and a C-terminal domain (CTD). The NTD adopts a typical spiral crotonase fold which is organized by two perpendicular β-sheets. The two perpendicular β-sheets contain six and two β-strands, respectively (**Figure 5D**). The CTD without the last 23 residues only contains two α helices (**Figure 5D**). The last 23 residues of the CTD missing in our structure probably form another helix according to the conservative analysis and structure alignment (**Figure 5E**). Thus, the complete structure of the CTD of *Rd*AcuH probably contains three α helices. There are three active centers in each trimer and each active center is located between the NTD of a subunit and the CTD of the neighboring subunit (**Figure 5E**).

### Analysis of the Key Residues Involved in the Catalysis of *Rd*AcuH

**FIGURE 6:**
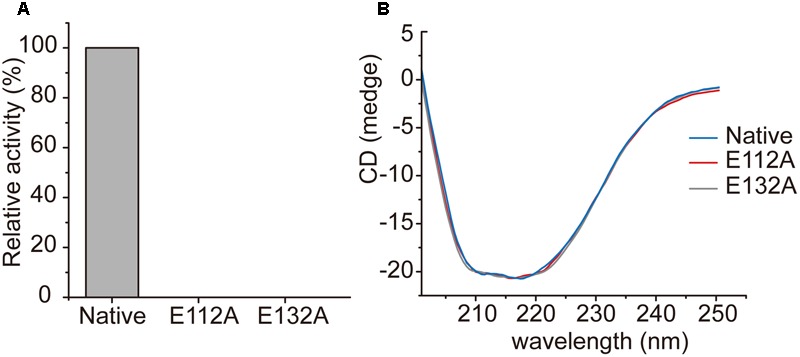
Characterization of *Rd*AcuH mutants. **(A)** The relative activity of *Rd*AcuH and its mutants. The activity of the wild type *Rd*AcuH was taken as 100%. No activity was detected for neither Glu112 mutant nor Glu132 mutant. **(B)** CD spectra of *Rd*AcuH and its mutants. The spectral curves of Glu112 mutant, Glu132 mutant and wild type *Rd*AcuH are almost identical, indicating that their secondary structures have little change.

In the structure of the enoyl-CoA hydratase from *R. norvegicus*, two conserved residues, Glu144 and Glu164, are close to the water molecule and the bounded substrate DAC-CoA molecule (Bahnson et al., 2002). Because *Rd*AcuH and the enoyl-CoA hydratase from *R. norvegicus* have high similarity in their sequences and structures, we found that the corresponding residues of these two glutamates in *Rd*AcuH are Glu112 and Glu132 according to the sequence and structure alignment (**Figures 2, 5E**). Draw on the experience of the enoyl-CoA hydratase from *R. norvegicus*, these two residues are probably also essential in the catalytic process of *Rd*AcuH. To confirm this, site-directed mutagenesis on these two residues were performed. Biochemical analysis showed that mutations on each of the glutamates to alanine resulted in the inactivation of *Rd*AcuH (**Figure 6A**). CD spectral analysis showed the site-directed mutations caused little change in the secondary structure of *Rd*AcuH (**Figure 6B**), indicating that the loss of enzyme activity was caused by the residue substitution rather than structural change. Thus, we suggest that Glu112 and Glu132 play important roles in the catalytic reaction of *Rd*AcuH, like the function of Glu144 and Glu164 in the enoyl-CoA hydratase from *R. norvegicus*.

## Discussion

AcuH is likely very important in the DMSP catabolism in bacteria because it is capable to participate in both the demethylation pathway and the cleavage pathway. In the demethylation pathway, AcuH is able to function as an isoenzyme of DmdD to catalyze methylthioacryloyl-CoA (MTA-CoA) hydration (Sullivan et al., 2011; Reisch et al., 2013; Bullock et al., 2017). In the cleavage pathway, it is involved in acrylate catabolism via catalyzing acryloyl-CoA hydration. Acrylate and acryloyl-CoA are all poisonous intermediate products during the metabolism of DMSP, and thus should be metabolized quickly in DMSP-catabolizing bacteria. Therefore, the involvement of AcuH in the metabolism pathway of acrylate is important for the detoxification of acrylate and acryloyl-CoA in DMSP-catabolizing bacteria.

**FIGURE 7:**
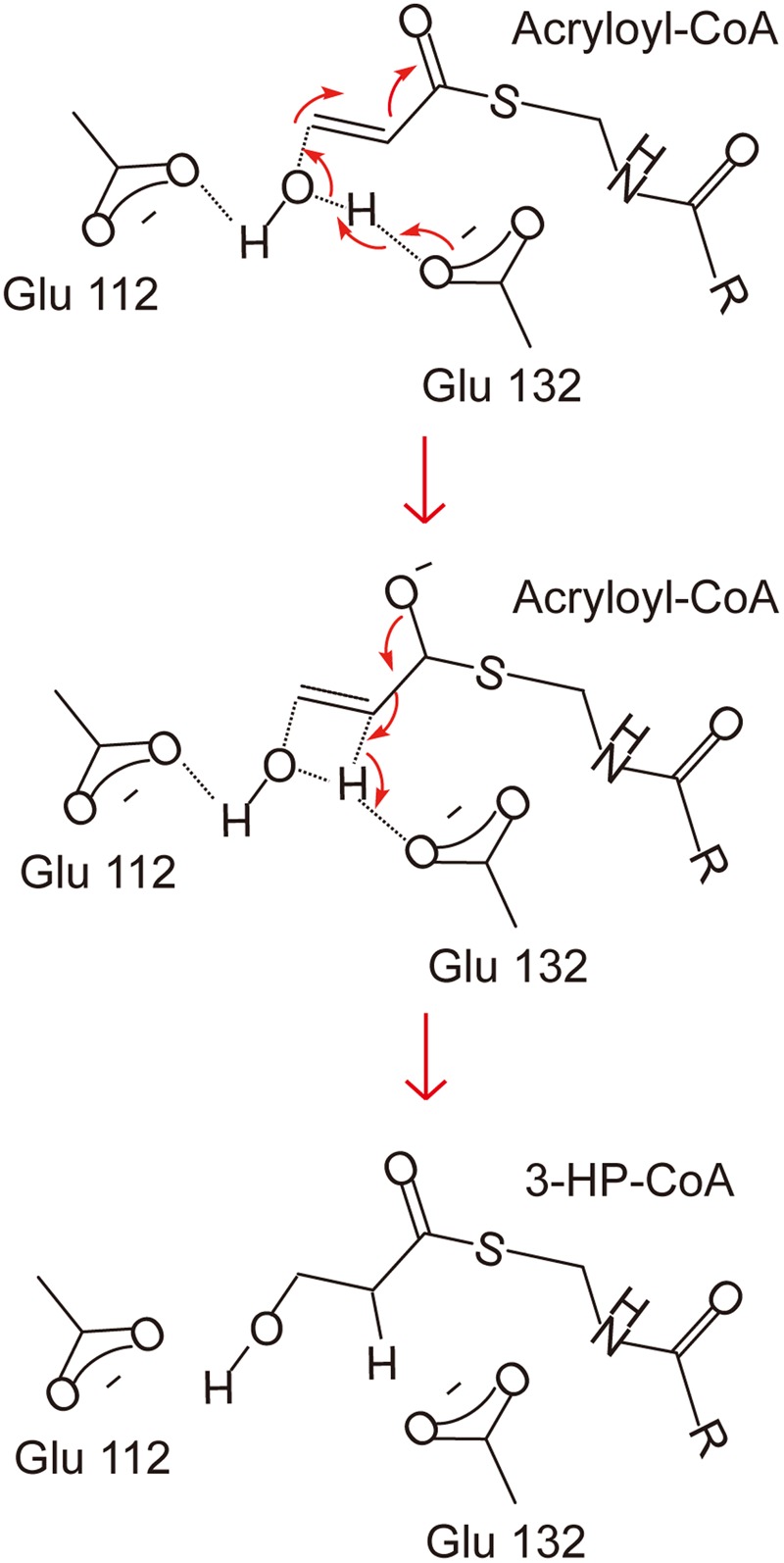
A proposed catalytic mechanism of *Rd*AcuH. The two residues, Glu112 and Glu132, play important roles in the reaction. They would be ionized under a neutral condition. Firstly, Glu132 acts as the brønsted base and transfers an electron to the water molecule. Then the activated water molecule attacks the C_3_ atom of acryloyl-CoA and the transition state is formed. Finally, the redundant electron is transferred to Glu132, the water molecule is added to acryloyl-CoA and 3HP-CoA is produced.

There has been no report on the property, structure or catalytic mechanism of AcuH from a DMSP catabolizing bacterium. In this study, we confirmed that the AcuH (*Rd*AcuH) from *Roseovarius nubinhibens* ISM, a DMSP catabolizing bacterium, has acryloyl-CoA hydration activity *in vitro*. Then we determined the structure of *Rd*AcuH. Due to the high similarity of *Rd*AcuH and the enoyl-CoA hydratase from *R. norvegicus* in structure, and similar conserved residues involved in catalysis, they likely have similar catalytic mechanism. The molecular mechanism of the enoyl-CoA hydratase from *R. norvegicus* for catalysis has been reported (Bahnson et al., 2002). Therefore, based on our structural and site-directed mutagenesis analyses, the catalytic mechanism for *Rd*AcuH could be deduced. Like the enoyl-CoA hydratase from *R. norvegicus*, there should be a water molecule in the position of the acetic acid molecule that acts as a catalytic water, and the carboxylates of Glu112 and Glu132 are expected to be ionized under a neutral condition. When an acryloyl-CoA molecule enters the active center, Glu132 would attack the water molecule and transfer an electron to it. Then the activated water molecule attacks the C_3_ atom of acryloyl-CoA and forms the transition state with the double bond. After that, acryloyl-CoA transmits the redundant electron to Glu132, and the water molecule is added to the acryloyl-CoA, leading to the production of 3HP-CoA (**Figure 7**). Then, 3HP-CoA is released and the *Rd*AcuH molecule is ready to catalyze the hydration of another acryloyl-CoA molecule. The elucidation of the catalytic mechanism of AcuH to hydrate acryloyl-CoA would shed light on the mechanism of acrylate detoxification in DMSP catabolizing bacteria.

Enoyl-CoA hydratases have a broad substrate specificity and can use enoyl-CoA of different lengths as substrates. For example, bovine liver enoyl-CoA hydratase can use CoA derivatives from crotonyl-CoA (4 carbons of the acyl chain) to 2-hexadecenoyl-CoA (16 carbons of the acyl chain) as substrates and the hydration rate (*V*_max_) decreases markedly with increasing chain length of the substrates (Waterson and Hill, 1972). Similarly, ECHS1 from human can also hydrate C4-C12 enoyl-CoA and has the highest activity for crotonyl-CoA (Yamada et al., 2015). These evidence suggests that the activity of enoyl-CoA hydratases increase with decreasing acyl chain length of the substrates. As an enoyl-CoA hydratase, *Rd*AcuH may also accept various substrates with different acyl chain length. Because acryloyl-CoA has only 3 carbons of the acyl chain, *Rd*AcuH should have a pretty high hydration rate for acryloyl-CoA, corresponding to its detoxification function in the acrylate metabolism pathway in DMSP catabolizing bacteria, which, however, still needs further confirmation.

Acryloyl-CoA can also be produced in other metabolic pathways in addition to DMSP metabolism pathway. For example, acryloyl-CoA in liver can be produced from 5,6-dichloro-4thia-5-hexenoyl-CoA (DCTH-CoA) by medium-chain acyl-CoA dehydrogenase or be produced from 4-(2-benzothiazole)-4-thiabutanoyl-CoA (BTTB-CoA) by the enoyl-CoA hydratase from *R. norvegicus* (Fitzsimmons et al., 1995; Baker-Malcolm et al., 2000). In addition, AcuH and its homologues are widespread (Bullock et al., 2017; Wang et al., 2017). Thus, the acryloyl-CoA detoxification mechanism by AcuH may be extended to other metabolic processes and have a wider significance.

## Author Contributions

H-YC and PW contributed equally to this work. X-LC, C-YL, and Y-ZZ designed research. H-YC and PW performed research. FX, P-YL, B-BX, Q-LQ, and Y-ZZ analyzed data. H-YC, PW, and X-LC wrote the paper.

## Conflict of Interest Statement

The authors declare that the research was conducted in the absence of any commercial or financial relationships that could be construed as a potential conflict of interest.

## Footnotes

1 https://www.dsmz.de/catalogues/details/culture/DSM-15170.html

2 http://www.pymol.org/

3 http://web.expasy.org/compute_pi/

4 http://www.cbs.dtu.dk/services/SignalP-4.0/

5 http://www.rcsb.org
